# Fluid intake-related association between urine output and mortality in acute respiratory distress syndrome

**DOI:** 10.1186/s12931-020-1286-5

**Published:** 2020-01-14

**Authors:** Yanfei Shen, Guolong Cai, Shangzhong Chen, Caibao Hu, Jing Yan

**Affiliations:** Department of Intensive Care, Zhejiang Hospital, No. 12, Linyin Road, Hangzhou, Zhejiang 310000 People’s Republic of China

**Keywords:** Acute respiratory distress syndrome, Fluid management, Mortality, Urine output, Fluid intake

## Abstract

**Background:**

Acute respiratory distress syndrome (ARDS), a complex response to various insults, has a high mortality rate. As pulmonary edema resulting from increased vascular permeability is a hallmark of ARDS, management of the fluid status, including the urine output (UO) and fluid intake (FI), is essential. However, the relationships between UO, FI, and mortality in ARDS remain unclear. This retrospective study aimed to investigate the interactive associations among UO, FI, and mortality in ARDS.

**Methods:**

This was a secondary analysis of a prospective randomized controlled trial performed at 10 centers within the ARDS Network of the National Heart, Lung, and Blood Institute research network. The total UO and FI volumes within the 24-h period preceding the trial, the UO to FI ratio (UO/FI), demographic data, biochemical measurements, and other variables from 835 patients with ARDS, 539 survivors, and 296 non-survivors, were analyzed. The associations among UO, FI, the UO/FI, and mortality were assessed using a multivariable logistic regression.

**Results:**

In all 835 patients, an increased UO was significantly associated with decreased mortality when used as a continuous variable (odds ratio [OR]: 0.98, 95% confidence interval [CI]: 0.98–0.99, *P* = 0.002) and as a quartile variable (OR of Q2 to Q4: 0.69–0.46, with Q1 as reference). To explore the interaction between UO and FI, the UO/FI was calculated, and a cut-off value of 0.5 was detected for the association with mortality. For patients with a UO/FI ≤0.5, an increased UO/FI was significantly associated with decreased mortality (OR: 0.09, 95% CI: 0.03–0.253, *P* <  0.001); this association was not significant for patients with UO/FI ratios > 0.5 (OR: 1.04, 95% CI: 0.96–1.14, *P* = 0.281). A significant interaction was observed between UO and the UO/FI. The association between UO and mortality was significant in the subgroup with a UO/FI ≤0.5 (OR: 0.97, 95% CI: 0.96–0.99, *P* = 0.006), but not in the subgroup with a UO/FI > 0.5.

**Conclusions:**

The association between UO and mortality was mediated by the UO/FI status, as only patients with low UO/FI ratios benefitted from a higher UO.

## Background

Acute respiratory distress syndrome (ARDS) is a complex response to pulmonary and non-pulmonary insults. This condition, which presents as severe hypoxemia and bilateral pulmonary infiltration, is associated with mortality rates of 30–40% [1, 2]. As pulmonary edema resulting from increased vascular permeability is a hallmark of ARDS [3], optimizing the fluid status is a fundamental concern in critical care practice.

Aggressive fluid resuscitation plays an important role in avoiding additional hemodynamic insults and maintaining adequate organ perfusion. However, considerable evidence indicates that a positive fluid balance (FB) may cause the extravasation of protein-rich fluids into the interstitial space and is strongly associated with poor outcomes in patients with ARDS [4–6]. However, most of these studies focused mainly on the absolute FB volume, which may have led to biased conclusions. For instance, hypothetical patient A (fluid intake 2000 ml, fluid output 1000 ml) and patient B (fluid intake 5000 ml, fluid output 4000 ml) might have the same absolute FB volume but very different outcomes. Therefore, a new index that could better reflect the dynamic fluid status has become clinically important.

Two intervenable parameters in fluid management, the fluid intake (FI) [7] and urine output (UO) values [8], have been investigated. Multiple observational studies involving different cohorts have reported that an increased UO volume was independently associated with decreased mortality [9, 10]. A multicenter randomized controlled trial that compared conservative and liberal fluid strategies in patients with ARDS also reported that loop diuretics were more frequently used and the UO volume was higher in the conservative fluid group [7]. In that trial, the conservative fluid strategy was associated with improved lung function and a reduced duration of mechanical ventilation. However, the correlation between UO and the outcomes of patients with ARDS remains unclear.

Furthermore, the UO volume may be easily affected by the FI volume. Therefore, a simple evaluation of the association between UO and mortality that does not adjust for FI may be inappropriate. Here, we created a new index (UO/FI ratio) to reflect the dynamic fluid status and performed this secondary analysis to investigate the interactive associations among UO, FI, and mortality in patients with ARDS.

## Methods

### Data source

This was a secondary analysis of a prospective RCT that was performed in 10 centers within the ARDS Network of the National Heart, Lung, and Blood Institute research network [11]. The original study was approved by the institutional review board at each study center, and informed consent was obtained from the patients or their legal guardians. All data were uploaded to the Biologic Specimen and Data Repository Information Coordinating Center (BioLINCC) (https://biolincc.nhlbi.nih.gov) by the ARDS Network. The re-use of these data for a retrospective study was approved by the institutional review board at each center and by BioLINCC, and the need for consent was waived.

### Inclusion and exclusion criteria

In the original study, patients under invasive mechanical ventilation support were screened if they met the following Berlin inclusion criteria [12]: an acute decrease in the ratio of the partial pressure of arterial oxygen to the fraction of inspired oxygen to ≤300, the presence of bilateral pulmonary infiltrates on chest radiography images, and no clinical evidence of left atrial hypertension or a pulmonary capillary wedge pressure of ≤8 mmHg. Patients were excluded if they were aged < 18 years, were pregnant, or had other clinical conditions that could impair breathing, such as high intracranial pressure. Patients without fluid management records were also excluded.

### Data extraction

The total UO and FI volumes within the 24-h period preceding the trial were recorded. Demographic data, including age, weight, height, sex, and ethnicity, and information regarding comorbidities such as diabetes, immunosuppression, and leukemia were collected. Biochemical measurements, including the white blood cell and platelet counts; serum creatinine, albumin, sodium, and bilirubin concentrations; and the plasma glucose concentration were also extracted. Other variables, such as the radiographic acute lung injury score, pneumothorax, and ratio of the partial pressure of arterial oxygen to the fraction of inspired oxygen, were recorded.

### Study endpoint

In the original study, patients were divided into three categories based on the following endpoints: (1) discharge with unassisted breathing, (2) death before discharge with unassisted breathing or before achieving unassisted breathing for 48 h, and (3) neither of these conditions. The patients’ statuses were checked at intervals of ≤30 days until either condition 1 or 2 was met, with a maximum duration of 180 days. Patients who met condition 2 were reported as non-survivors, whereas those who met condition 1 or 3 were reported as survivors.

### UO/FI ratio

The UO/FI ratio was calculated to assess the ability to excrete excessive administered fluid. The association between the UO/FI ratio and mortality was evaluated.

### Missing data management

For most of the extracted variables, the proportion of missing values was < 5%, and these values were replaced by their means or medians. For albumin, the proportion of missing values was > 10%; therefore, this variable was excluded from the analysis. For dichotomous variables (diagnosis such as diabetes), missing values were replaced by default value (zero).

### Statistical analysis

Continuous variables are expressed as means ± standard deviations or medians (interquartile ranges) as appropriate. Student’s t-test and the Wilcoxon rank-sum test were used as appropriate. Categorical data are expressed as proportions and were compared using the chi-squared test or Fisher’s exact test.

A multivariable logistic regression was used for covariate adjustment. The logistic models were built using the stepwise backward method. First, variables with *P*-values of < 0.10 in the univariate analyses were included in the multivariable analysis. Twelve covariables were identified in this step: UO, low tidal volume intervention, leukemia, solid tumor, immune suppression, body temperature, mean blood pressure, respiratory rate, hematocrit, platelet count, serum bicarbonate, radiographic acute lung injury score, and the ratio of the partial pressure of arterial oxygen to the fraction of inspired oxygen on day 0. Subsequently, a stepwise backward elimination method was used to remove variables with *P*-values > 0.05 (serum bicarbonate and immune suppression). Multicollinearity was assessed using the variance inflation factor method, and body temperature, hematocrit, and mean blood pressure were removed as significant variance inflation factors (≥5).

The Lowess smoothing technique was used to explore the crude relationship between the UO/FI ratio and mortality, and a cut-off value was detected. A spline linear logistic regression analysis was performed to evaluate the association between the UO/FI ratio and mortality, using the UO/FI ratio cut-off value. The interaction between UO and the UO/FI ratio was evaluated by adding the interactive item in the logistic model, and a subgroup analysis was conducted. The predictive marginal effects of the UO were estimated for different UO/FI ratios. A two-tailed test was performed, and a *P*-value < 0.05 was considered to reflect statistical significance. All statistical analyses were performed using Stata 11.2 (StataCorp, College Station, TX, USA).

## Results

The data of 902 patients were available in the dataset downloaded from BioLINCC. Sixty-seven patients were excluded because of a lack of relevant records. Thus, 539 survivors and 296 non-survivors (835 total patients) were included in the final analysis. The overall mortality rate was 35.4%. Compared to non-survivors, survivors had a significantly higher UO volume within the 24-h period preceding the trial (27.9 ± 23.4 vs. 33.2 ± 25.8 mL/24 h, *P* = 0.003) and significantly lower FI volume (71.4 ± 56.8 vs. 63.4 ± 52.5 mL/24 h, *P* = 0.039). The maximum serum creatinine concentration was similar between survivors and non-survivors (1.59 ± 1.54 vs. 1.80 ± 1.47 mmol/L, *P* = 0.058). The detailed baseline characteristics and comparisons are listed in Table 1.

**Table 1:** Comparisons of baseline characteristics between survivors and non-survivors

Variables	Overall (*n* = 835)	Survivors (*n* = 539)	Non-survivors (*n* = 296)	*P*
Age (years)	51.1 ± 16.4	47.8 ± 16.5	59.1 ± 16.4	< 0.001
Male [n (%)]	494 (59.1)	314 (58.2)	180 (60.8)	0.472
Height [n (%)]	171.2 ± 9.7	171.8 ± 9.7	170.2 ± 9.5	0.021
Weight [n (%)]	79.7 ± 21.6	80.5 ± 22.0	78.2 ± 20.9	0.134
Ethnicity (Black, %)	148 (17.7)	90 (16.6)	58 (19.5)	0.294
Ethnicity (white, %)	606 (74.9)	401 (74.3)	205 (69.2)	0.111
Low tidal volume intervention [n (%)]	443 (53.0)	304 (56.4)	139 (46.9)	0.009
Comorbidities				
Leukemia [n (%)]	17 (2.0)	5 (0.9)	12 (4.0)	0.004
Immunosuppression [n (%)]	86 (10.2)	41 (7.6)	45 (15.2)	0.001
Diabetes [n (%)]	118 (14.1)	72 (13.5)	46 (15.5)	0.386
Solid tumour [n (%)]	16 (1.9)	6 (1.1)	10 (3.4)	0.032
Lymphoma [n (%)]	9 (1.1)	3 (0.5)	6 (2.0)	0.075
Cirrhosis [n (%)]	24 (2.8)	12 (2.2)	12 (4.0)	0.136
Elective surgery [n (%)]	73 (8.7)	46 (8.5)	27 (9.1)	0.774
Pneumothoraces [n (%)]	105 (12.5)	68 (12.6)	37 (12.6)	0.961
Chest tube [n (%)]	210 (25.1)	140 (25.9)	70 (23.6)	0.459
Biochemical indexes				
PaO2/FiO2 (mmHg)	150.9 ± 69.8	156.4 ± 71.2	141.0 ± 66.0	0.002
Maximum respiratory rate	30.4 ± 10.8	29.7 ± 11.1	31.7 ± 10.2	0.011
Maximum mean blood pressure (mmHg)	101.9 ± 19.7	103.3 ± 19.9	99.3 ± 19.2	0.005
Minimum mean blood pressure (mmHg)	61.8 ± 13.6	63.4 ± 13.8	59.0 ± 12.8	< 0.001
Maximum white blood cell (10^9/L)	14.9 ± 10.2	15.0 ± 9.9	14.7 ± 10.6	0.672
Minimum white blood cell (10^9/L)	12.1 ± 9.0	12.3 ± 8.9	11.7 ± 9.3	0.366
Minimum platelet count (10^9/L)	161.7 ± 116.7	169.7 ± 121.2	147.3 ± 106.8	0.008
Maximum serum creatinine (mg/L)	1.67 ± 1.52	1.59 ± 1.54	1.80 ± 1.47	0.058
Minimum serum sodium (mmol/L)	136.9 ± 5.5	136.9 ± 5.2	136.9 ± 6.1	0.995
Maximum serum sodium (mmol/L)	139.3 ± 5.3	139.2 ± 4.9	139.3 ± 6.0	0.762
Minimum serum albumin (g/dl)	2.19 ± 0.57 (*n* = 726)	2.26 ± 0.58 (*n* = 469)	2.08 ± 0.54 (*n* = 257)	< 0.001
Minimum serum bicarbonate (mmol/L)	21.4 ± 5.4	21.9 ± 5.4	20.6 ± 5.1	0.001
Fluid records				
Fluid intake (ml/kg/24 h)	66.2 ± 54.2	63.4 ± 52.5	71.4 ± 56.8	0.039
Urine output (ml/kg/24 h)	31.3 ± 25.1	33.2 ± 25.8	27.9 ± 23.4	0.003
UO /FI	0.75 ± 1.82	0.76 ± 0.92	0.72 ± 2.80	0.777

*Abbreviation*: *UO/FI* Urine output/fluid intake

### Association between UO and mortality

**Fig. 1:**
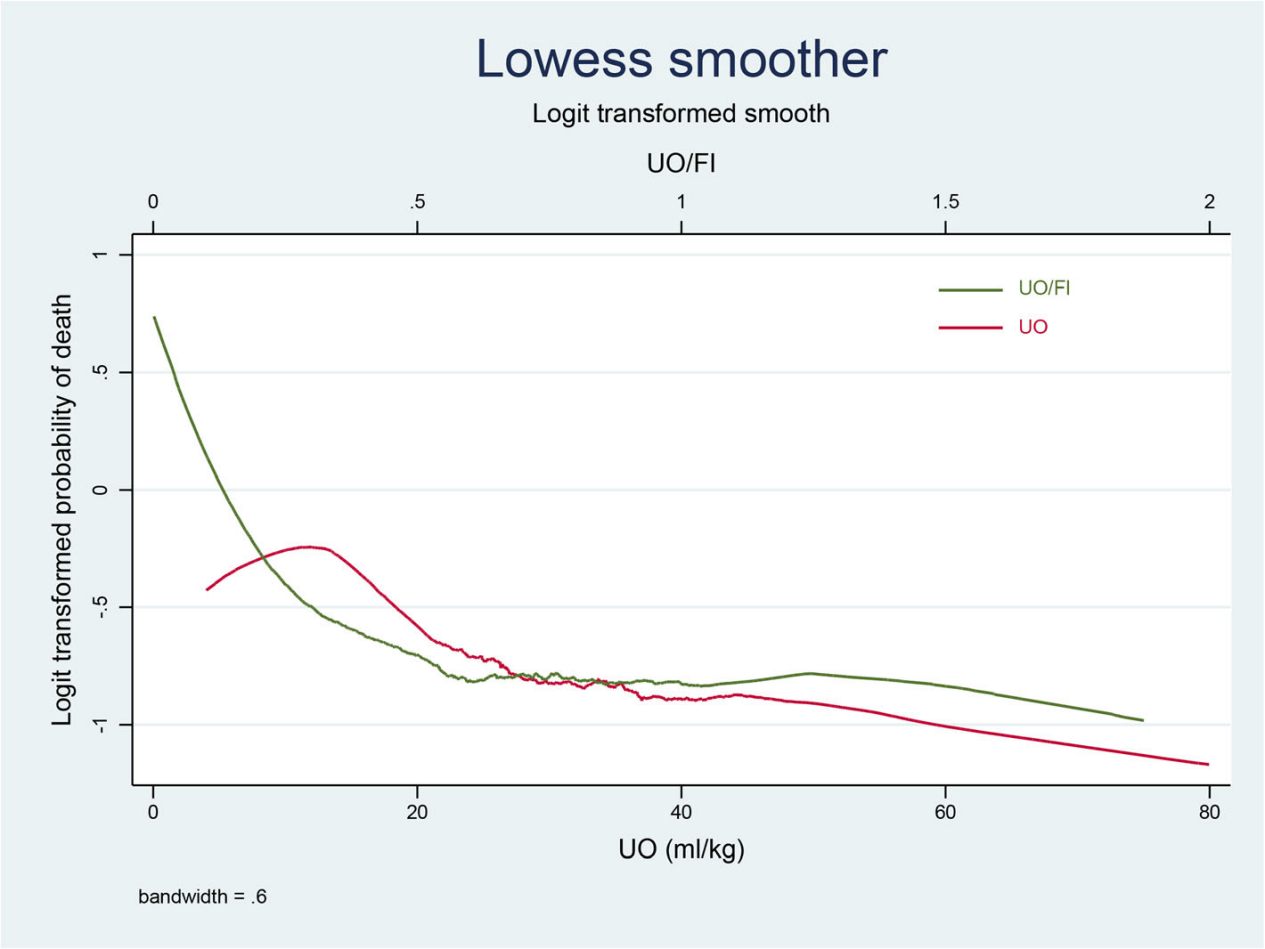
Associations among the urine output (UO), urine output/fluid intake (FI) ratio, and hospital mortality in patients with acute respiratory distress syndrome. A turning point of green curve around 0.5 was observed, top horizontal axis

A multivariable logistic analysis was used to explore the adjusted association between UO and mortality (Table 2). For maximum statistical efficiency, UO was included as a continuous variable in model 1 (Table 2), and the odds ratio (OR) was significant (OR: 0.98, 95% confidence interval [CI]: 0.98–0.99, *P* = 0.002). For interpretation, UO was used as a quartile variable in model 2 (Table 2), and a stepwise decreasing trend was observed from quartile two (OR: 0.69, 95% CI: 0.46–1.03, *P* = 0.072) to quartile four (OR: 0.46, 95% CI: 0.30–0.70, *P* <  0.001) relative to quartile one. The trend of the curve in Fig. 1 is consistent with the abovementioned findings.

**Table 2:** Two multivariable logistic models using UO as continuous and dummy variables

Model 1			Model 2		
Variables	Adjusted odds ratio (95% CI)	*P*	Variables	Adjusted odds ratio (95% CI)	*P*
UO	0.98 (0.98–0.99)	0.002	UO quartile 1	Ref.	
Low tidal volume intervention	0.67 (0.50–0.90)	0.009	UO quartile 2	0.69 (0.46–1.03)	0.072
Leukemia	3.95 (1.31–11.8)	0.014	UO quartile 3	0.51 (0.34–0.78)	0.002
Solid tumour	3.26 (1.14–9.34)	0.027	UO quartile 4	0.46 (0.30–0.70)	< 0.001
Respiratory rate	1.01 (1.00–1.03)	0.015	Low tidal volume intervention	0.68 (0.51–0.92)	0.013
Platelet count (10^9/L)	0.99 (0.99–0.99)	0.004	Leukemia	3.26 (1.09–9.73)	0.033
PaO_2_/FiO_2_	0.99 (0.99–0.99)	0.003	Solid tumour	3.57 (1.24–10.25)	0.018
			Respiratory rate	1.01 (1.00–1.03)	0.013
			Platelet count (10^9/L)	0.99 (0.99–0.99)	0.005
			PaO2/FiO2	0.99 (0.99–0.99)	0.004

UO was used as a continuous variable in Model 1 and was divided into four quartiles in Model 2. The VIF value were 2.63 and 2.46 for Model 1 and Model 2, respectively

*Abbreviation*: *UO* Urine output

### Association between UO/FI and mortality

The crude relationship between the UO/FI ratio and mortality was explored using the Lowess smoothing technique, as shown in in Fig. 1. A cut-off value of 0.5 was determined and applied in the multivariable linear spline logistic regression (Table 3). Among subjects with UO/FI ratios ≤0.5, an increased UO/FI ratio was significantly associated with decreased mortality (OR: 0.09, 95% CI: 0.03–0.253, *P* <  0.001); however, this association was not significant for UO/FI ratios > 0.5 (OR: 1.04, 95% CI: 0.96–1.14, *P* = 0.281).

**Table 3:** Linear spline associations between UO/FI and mortality

Variables	Crude odds ratio (95% CI)	*P*	Adjusted odds ratio (95% CI)	*P*
UO/FI ≤ 0.5	0.08 (0.03–0.22)	< 0.001	0.09 (0.03–0.25)	< 0.001
UO/FI > 0.5	1.04 (0.95–1.13)	0.353	1.04 (0.96–1.14)	0.281
Low tidal volume intervention			0.68 (0.51–0.92)	0.013
Leukemia			3.56 (1.19–10.6)	0.022
Solid tumour			3.38 (1.18–9.67)	0.023
Respiratory rate			1.01 (1.00–1.03)	0.013
Platelet count (10^9/L)			0.99 (0.99–0.99)	0.027
PaO_2_/FiO_2_			0.99 (0.99–0.99)	0.005

Linear spline function was applied in the logistic models using cut-off value of 0.5 of UO/FI. The VIF value was 3.03 in the multivariable logistic model

*Abbreviation*: *UO/FI* Urine output/fluid intake

### Interaction between UO and UO/FI

**Fig. 2:**
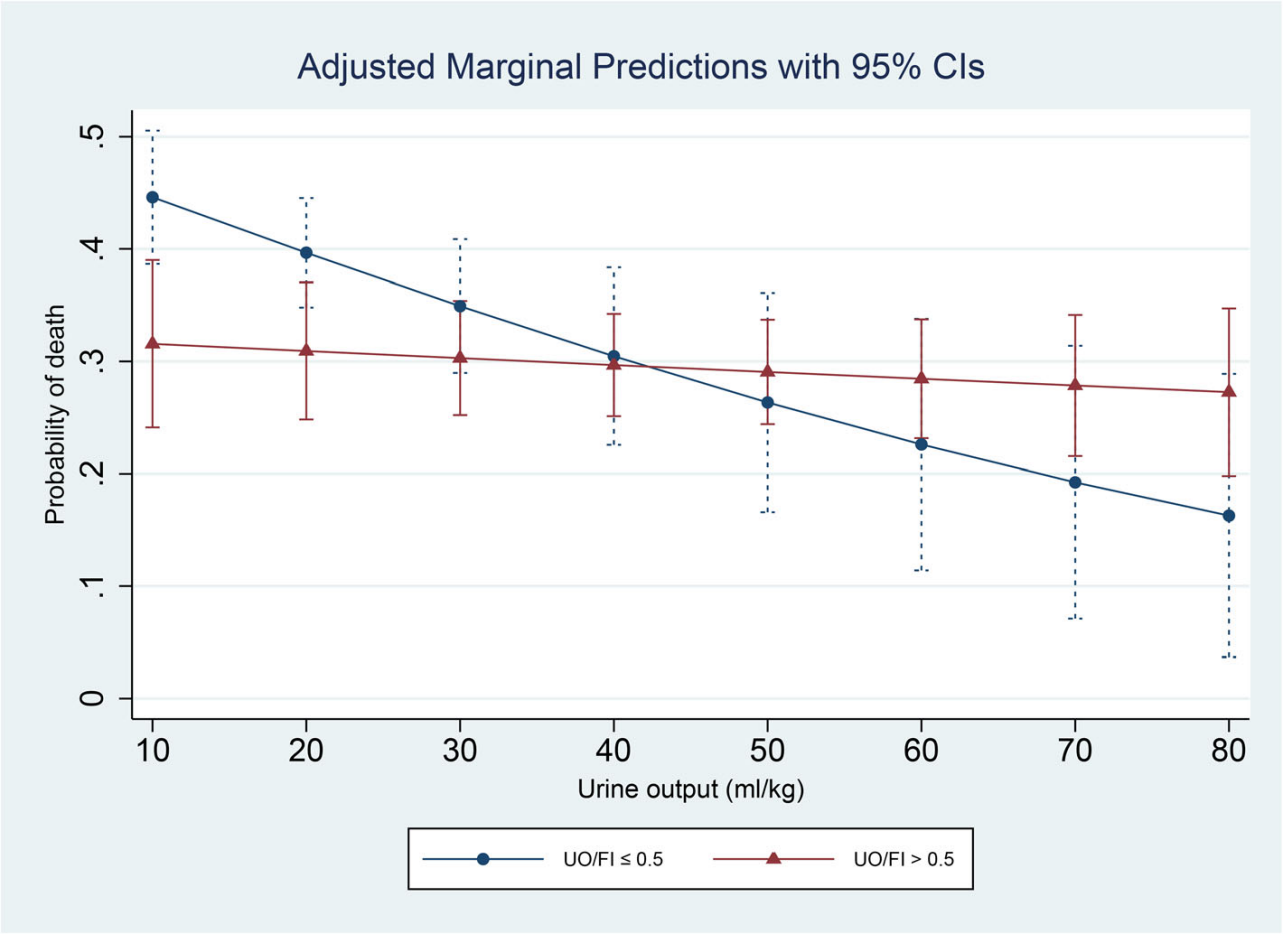
The predictive marginal effect of urine output in patients with different urine output (UO)/fluid intake (FI) ratios

The interaction between UO and the UO/FI ratio was significant when the UO/FI ratio was used as a dummy variable (≤0.5 or > 0.5, *P*-value for the interaction = 0.044; see table in Additional file 1). A subgroup analysis was conducted using this UO/FI ratio cut-off value. The association between UO and mortality was significant in the subgroup with UO/FI ratios ≤0.5 (Table 4, OR: 0.97, 95% CI: 0.96–0.99, *P* = 0.006), but was not significant in the subgroup with UO/FI ratios > 0.5 (OR: 0.99, 95% CI: 0.98–1.01, *P* = 0.504). The predictive marginal effects of different UO values (10, 20, 30, 40, 50, 60, and 70 mL/kg/24 h) on mortality were also estimated at different UO/FI values (≤0.5 or > 0.5) as shown in Fig. 2. The slope of the predictive curve between UO and mortality at a UO/FI ratio ≤ 0.5 was markedly steeper than the slope of the corresponding curve at a UO/FI ratio > 0.5, consistent with the above findings.

**Table 4:** Subgroup analysis of patients with high and low UO/FI ratios

Subgroup with UO/FI ≤ 0.5 (Model A, *n* = 421)			Subgroup with UO/FI > 0.5 (Model B, *n* = 414)		
Variables	Adjusted odds ratio (95% CI)	*P*	Variables	Adjusted odds ratio (95% CI)	*P*
UO	0.97 (0.96–0.99)	0.006	UO	0.99 (0.98–1.00)	0.504
Low tidal volume intervention	0.80 (0.53–1.20)	0.295	Low tidal volume intervention	0.57 (0.36–0.88)	0.013
Leukemia	5.83 (1.17–29.1)	0.031	Leukemia	1.92 (0.35–10.39)	0.447
Solid tumour	2.15 (0.42–10.82)	0.351	Solid tumour	4.21 (1.00–17.50)	0.048
Respiratory rate	1.01 (0.99–1.03)	0.165	Respiratory rate	1.02 (1.00–1.04)	0.034
Platelet count (10^9/L)	0.99 (0.99–1.00)	0.161	Platelet count (10^9/L)	0.99 (0.99–0.99)	0.032
PaO_2_/FiO_2_	0.99 (0.99–0.99)	0.039	PaO2/FiO2	0.99 (0.99–0.99)	0.039

The *p* value for interaction between UO and UO/FI ratio was 0.044. The VIF value were 2.69 and 2.83 for Model A and Model B, respectively

*Abbreviations*: *UO* Urine output, *UO/FI* Urine output/fluid intake

## Discussion

With this study, we aimed mainly to direct attention to the dynamic fluid status rather than the absolute fluid management volume. Here, we used the UO/FI ratio to reflect a patient’s ability to excrete excess administered fluids. We determined that for patients with a UO/FI ≤0.5, an increase in the UO/FI was significantly associated with decreased mortality. However, this association was non-significant for those with a UO/FI > 0.5. Furthermore, we also found that the UO/FI ratio significantly influenced the association between UO and mortality in ARDS. Moreover, the association between UO and mortality was significant only among patients with a UO/FI ratio ≤ 0.5. Therefore, this study offers novel insights into the complex interactions among UO, FI, and mortality in ARDS.

Appropriate fluid management is critical to the overall management of patients with ARDS, as pulmonary edema resulting from increased capillary permeability is a characteristic feature [3]. Several retrospective studies [5, 6] reported that both early and late increased fluid accumulation are significantly associated with poor outcomes in ARDS. Accordingly, recent investigations have focused on strategies to limit FI and increase the fluid output, with the aim of alleviating these poor outcomes [7, 13, 14].

In 2006 [7], the ARDS Network compared the efficacy of liberal and conservative fluid strategies and found that conservative fluid administration (more loop diuretics and higher UO volume) was shown to improve lung function and reduce the duration of mechanical ventilation. In another RCT, Martin et al. [15] found that when compared to a placebo, albumin and furosemide combination therapy resulted in a significantly higher UO during the intervention period, which was associated with an improved fluid balance, oxygenation, and hemodynamics in hypoproteinemic patients with acute lung injury. However, as both the diuretic [14, 15] and non-diuretic [16, 17] effects of furosemide may be responsible for the improved outcomes, the direct association between UO and mortality in ARDS cannot be inferred from these trials.

Multiple observational studies have demonstrated an independent association of increased UO with decreased mortality in unselected critically ill patients [9] or in patients with acute kidney injury [10]. In an observational study of 81 patients with ARDS who were receiving extracorporeal membrane oxygenation support, Hsiao et al. [18] found that the UO within the initial 24 h after the commencement of extracorporeal membrane oxygenation support, the mean arterial pressure, and the platelet count were independent risk factors for hospital mortality. Indeed, a decreased UO may indicate low renal perfusion and consequent fluid overload, which in turn contributes to subsequent organ dysfunction [8]. Nevertheless, it remains unclear whether these findings are translatable to regular ARDS patients, given the remarkable heterogeneity among these cohorts. Furthermore, a simple evaluation of the association between UO and mortality that is not adjusted for FI is insufficient. For instance, the clinical outcomes of two hypothetical patients with a similar UO volume of 1000 ml, depending on the FI.

In the current study, we observed a linear correlation between UO and the probability of hospital mortality. However, after adjusting for FI, we noticed that this association between UO and mortality was significant only in patients with low UO/FI ratios. We further identified a non-linear association between the UO/FI ratio and mortality. To some extent, these findings suggest a point of equilibrium between UO and FI, and also raise some new questions. For instance, would a patient with a low UO/FI ratio benefit from an increase in the fluid intake as guided by the UO/FI? Further, would the benefits of diuretics remain significant in patients with high UO/FI ratios? Of course, the underlying mechanisms, particularly with regard to the cut-off value, cannot be inferred due to the retrospective nature of this study. We speculate that the low UO/FI ratios imply some level of decompensatory organ function, such as cardiac failure, kidney failure, or unstable hemodynamics. However, the ability to excrete a greater UO volume may suggest relatively better organ function and less fluid accumulation, and may thus explain why a greater UO volume was associated with improved mortality. The cut-off value of 0.5 may serve as an indicator of the boundary between decompensatory and compensatory organ function and fluid accumulation. Hence, in patients with the ability to achieve high UO/FI ratios (> 0.5), the association between UO and mortality would not be significant. Further studies are needed to validate our hypothesis and re-evaluate the heterogeneous effects of fluid strategies, such as fluid restriction and diuretic use, in patients with different UO/FI ratios.

Our study had several advantages. First, all data were extracted from a rigorously designed multicenter trial, which guaranteed the accuracy of the data. Second, in contrast to previous studies, both UO and FI were used as continuous variables in our study (the OR was small, as it only represented the change in odds per UO or FI unit increase [1 mL/kg/24 h]), which maximized the statistical power. Third, to the best of our knowledge, this is the first study to report the UO/FI ratio, which may provide a better reflection of the fluid status than the UO alone. Further studies are needed to validate our findings.

Several limitations of our study should also be mentioned. First, we included as many potential confounders as possible, but could not exclude residual confounding bias. For instance, the interactive effects between diuretics and the UO/FI ratio could not be evaluated in this study because the dataset did not include diuretic records. Second, we only analyzed fluid records obtained within the 24-h period preceding the original trials. Therefore, it remains unclear whether the observed association would remain consistent across different time intervals. Third, the original study included only patients under invasive mechanical ventilation support, which restricts the applicability of our findings. Finally, the retrospective nature of the study limited our ability to determine a causal relationship between the UO volume and mortality. For instance, a higher UO volume may be an indicator of better kidney function, rather than a determinant of mortality. Thus, additional investigation is needed to determine whether strategies designed to increase the UO could improve the clinical outcomes of patients with ARDS.

## Conclusion

In conclusion, in patients with ARDS, an increased UO was associated with decreased mortality. However, this association was influenced by the UO/FI ratio, and was only significant in the subgroup with UO/FI ratios of ≤0.5. Further studies are needed to validate and expand our findings.

## Supplementary information

**Additional file 1: Table S1.** describing the interaction between UO and UO/FI.

## Data Availability

The datasets used and/or analyzed during the current study are available from YS on reasonable request (with permission of BioLINCC).

## Abbreviations

ARDS: Acute respiratory distress syndrome
BioLINCC: Biologic Specimen and Data Repository Information Coordinating Center
CI: Confidence interval
FI: Fluid intake
OR: Odds ratio
RCT: Randomized controlled trial
UO: Urine output
