# Supplementary material for: Fluid intake-related association between urine output and mortality in acute respiratory distress syndrome
Source: Respir Res. 2020 Jan 14;21:24. doi: 10.1186/s12931-020-1286-5 (PMC6961352; doi:10.1186/s12931-020-1286-5)
Supplement: Supplementary file 1 — Additional file 1: Table S1. describing the interaction between UO and UO/FI. [file 12931_2020_1286_MOESM1_ESM.doc]

Table S1. Interaction between UO and UO/FI

| **Variables** | **Adjusted odds ratio (95% CI)** | **p** |
| --- | --- | --- |
| **UO** | 0.97 (0.96 – 0.99) | 0.007 |
| **UO/FI ≤ 0.5** | 0.48 (0.28 – 0.82) | 0.008 |
| **UO * UO/FI (interaction item)** | 1.01 (1.00 – 1.03) | 0.044 |
| **Low tidal volume intervention** | 0.68 (0.51 – 0.92) | 0.014 |
| **Leukemia** | 3.60 (1.19 – 10.85) | 0.022 |
| **Solid tumor** | 3.42 (1.19 – 9.76) | 0.022 |
| **Respiratory rate** | 1.01 (1.00 – 1.03) | 0.016 |
| **Platelet count (10^9/L)** | 0.99 (0.99 – 0.99) | 0.010 |
| **PaO2/FiO2** | 0.99 (0.99 – 0.99) | 0.003 |

Note: The VIF value was 5.42 in the multivariable logistic model.

Abbreviations: UO, urine output; UO/FI, urine output/fluid intake; PaO_2_^/FiO^_2_, ratio of partial pressure of arterial oxygen to fraction of inspired oxygen
